# Trends in prevalent TB among persons enrolling for HIV care before and after ‘Test and Treat’ across East-Africa

**DOI:** 10.5588/ijtldopen.24.0687

**Published:** 2025-06-13

**Authors:** N. Kalema, B. Musick, S. Babirye, L. Najjemba, P Mubiri, A. Kiragga, A. Ddungu, C. Kasozi, L.O. Diero, F. Odhiambo, R. Lyamuya, B. Castelnuovo, J. Musaazi, C.T. Yiannoutsos, K. Wools-Kaloustian, A. Semeere

**Affiliations:** ^1^ Infectious Diseases Institute, Makerere University, Kampala, Uganda;; ^2^ Department of Medicine Indiana University School of Medicine, Indianapolis, Indiana;; ^3^ African Population and Health Research Center (APHRC), Nairobi, Kenya; USA;; ^4^ Masaka Regional Referral Hospital, Masaka, Uganda;; ^5^ AMPATH, Moi-University Referral Hospital, Eldoret, Kenya;; ^6^ Kenya Medical Research Center, Nairobi, Kenya;; ^7^ Morogoro Regional Referral Hospital, Tanzania.

**Keywords:** tuberculosis, prevalence, PLHIV, ART, Uganda, Kenya, Tanzania

## Abstract

**BACKGROUND:**

In 2015, WHO recommended the global adoption of the ‘Test and Treat’ strategy (TTS) for all persons living with HIV (PLHIV). While TTS has improved viral suppression and reduced mortality, its impact on TB in PLHIV remains unclear.

**METHODS:**

We assessed TB prevalence trends 48 months before and after TTS among PLHIV aged ≥18 years enrolling at HIV primary care sites affiliated with the East Africa International Epidemiology Databases to Evaluate AIDS (EA-IeDEA) consortium. We defined prevalent TB as bacteriologically confirmed or empirically treated TB within 60 days of enrolment. We estimated monthly TB prevalence trends using Poisson (change point) model.

**RESULTS:**

Among 125,647 PLHIV, 37% were male. The prevalence of TB was 8.9% (95% CI: 8.7–9.1) before and 6.2% (95% CI: 5.9–6.4) after TTS-adoption. Adjusted analysis showed significant downward trend in TB prevalence before TTS (adjusted Prevalence Rate Ratio, aPRR=0.989, p<0.001), which plateaued during TTS (aPRR=0.999, p=0.131). TB was more frequently present among males (aPRR: 2.09, p<0.001) and adults ≥25 years across both periods.

**CONCLUSION:**

This study highlights a plateau in TB prevalence decline during TTS and persistent disparities in TB by sex and age, underscoring the need for targeted interventions.

The regions of Eastern and Southern Africa account for 54% (between 18.9–23.0 million]) of the people living with HIV (PLHIV) and 71–74% of the HIV-TB co-infection burden (2011–2022) globally.^1,2,3^ Because TB is the leading cause of mortality among PLHIV, with a 40% higher risk of death with TB compared to those without, the prevalence of co-infection is of great concern. In 2023 alone, TB was responsible for a quarter of the 630,000 AIDS-related deaths globally.^4^ In the absence of antiretroviral therapy (ART), the risk of PLHIV developing active TB increases by 1.43-fold for every 100 cells/uL decrease in CD4 T-cell count.^5^ As such, early initiation of ART in PLHIV is critical for preventing progressive immune-suppression which predisposes one to the emergence of opportunistic infections, the most common being TB.^6,7^

In 2015, WHO recommended the ‘Test and Treat’ strategy (TTS) in which PLHIV initiate ART on the day they are diagnosed, to reduce morbidity and mortality associated with HIV/AIDS and its attendant opportunistic infections.^6–8^ Early ART initiation alone reduces the risk of TB disease in PLHIV by up to 65%^9–12^ However, systematic reviews of TB prevalence data between 1990–2019, reveal that the prevalence of HIV-TB co-infection in sub-Saharan Africa (SSA), particularly Eastern Africa (EA), has remained persistently high (ranging from 24–32%), before and after TTS.^1,13–16^

Despite the 2023 WHO Global TB Report indicating a decline of incident TB in Africa and globally, Uganda, Kenya and Tanzania, continue to be listed among the top 30 countries in the world with high TB-, and TB-HIV burden with a combined regional TB incidence of 219 (95% Uncertainty Interval: 151–288) cases per 100,000 population and an average HIV prevalence of 5% in 2022,^1,17–19^ 6 years after adopting TTS.^10^ The contrast between WHO reports of a declining TB burden in Africa and the persistently high TB burden observed in East African countries raises questions about the impact of TTS on TB prevalence trends, particularly among PLHIV, who face a high risk of TB co-infection. Estimating post-TTS TB burden in PLHIV will inform current and future End TB strategies aiming to lower incident TB by 90% in 2030 (from a 2015 baseline). We have therefore compared the trends in TB prevalence among PLHIV before and after the introduction of TTS in the EA Region.

## METHODS

We analysed cross-sectional de-identified patient-level data of adult PLHIV in the East Africa International Epidemiology Databases to Evaluate AIDS (EA-IeDEA) affiliated programs between January 2012 and December 2020.

Through its Regional Data Center (RDC), EA-IeDEA collects, harmonizes, and analyses data drawn from eight HIV care and treatment programs in Uganda, Kenya, and Tanzania. These include: in Kenya the Academic Model Providing Access to Healthcare (AMPATH) and the Kenya Medical Research Institute - Centre for Microbiology Research (KEMRI-CMR) – Research, Care and Training Program, Kisumu-Kenya; in Uganda the Infectious Diseases Institute (IDI), the Rakai Health Sciences Program, and Masaka Regional Referral hospital HIV clinic; and in Tanzania the Morogoro Regional Hospital, Tumbi Special Hospital, and Kisesa Health Centre Care and Treatment Clinics. (Supplementary Data Table S4) These programs followed country-specific Ministry of Health (MoH) guidelines, which were largely based on WHO guidelines for HIV care and treatment ^20^. These guidelines include guidance on TB screening and diagnosis, as well as ART initiation and monitoring. In general, across sites and over the study period, viral load monitoring was recommended at 6 and 12 months after ART initiation followed by annual monitoring if virally suppressed ^21-23^. PLHIV enrolling between January 2012 and December 2015, initiated ART only after their CD4 count had dropped below the MoH guideline-set threshold and/or if their WHO disease stage was above the prescribed threshold. In 2016, revised WHO guidelines recommended that all newly identified PLHIV start ART upon receiving a positive test result, regardless of their CD4 count or disease stage ^20^. All eight EA-IeDEA programs provided dates when TTS was initiated.

Medical records of PLHIV who were ≥18 years and enrolled in care at an EA-IeDEA-affiliated HIV care and treatment programs, 48 months before and after TTS adoption were included in this study. Patient-level data were either entered directly into the local electronic-medical-record by clinicians during the course of the patient visit or collected on structured paper-based forms, which were later entered into the electronic-medical-record by trained data assistants. De-identified data were transferred to the EA Regional Data Center in Indianapolis where program-specific master datasets were generated, harmonized and merged to create analysis datasets for this project. Data quality checks were incorporated into each step of the process.

The primary outcome of interest was prevalent TB disease at enrolment into an EA-IDEA-affiliated HIV care and treatment program. Prevalent TB status was determined as a positive diagnostic test (on microscopy, Xpert MTB/Rif, Urine TB LAM test or MTB culture) or a TB diagnosis recorded by the clinician within 60-days of enrolment into care. In the absence of a test or diagnostic information, provision of TB treatment within 60-days of enrolment into care was used to assign a positive TB status at enrolment. TB prevalence was computed as a proportion of subjects with a positive TB status out of all PLHIV enrolling into care, accompanied by 95% confidence intervals (CI). Descriptive statistics included frequencies, proportions for categorical variables and medians and interquartile ranges (IQR) for continuous variables. Pearson’s Chi-square test and Mann-Whitney U test were used to compare patient characteristics before and after TTS. We explored month-to-month changes in TB prevalence (trends) using interrupted time-series analysis. TB prevalence was estimated at each month as a proportion of prevalent TB cases over the total PLHIV enrolled in that month. Firstly, we plotted the observed TB prevalence rates over time to visualize the pattern of month-to-month changes in the TB prevalence before and after TTS roll-out. We attempted to assess the presence of a possible association between TB prevalence and time prior to and after initiation of TTS via a changepoint (CP) approach with TTS being a possible inflection point. A byproduct of this model is the ability to assess whether there was a different trend (slope of the trend line) in TB prevalence prior to and after the initiation of TTS. This model is expressed as follows:

$$\log\left(y_{i}\right)=\beta_{0}+\beta_{1}{t_{i}}^{-}+\beta_{2}{t_{i}}^{+}+\log\left(T_{i}\right)$$

Secondly, we fitted a Poisson regression model, with (the logarithm of) the total number of TB cases $y_{i}$ considered as the outcome and (the logarithm of) the total number of patients enrolling in care within each month $T_{i}$ as the offset adjusted for age, sex and program to account for clustering nature of our data and identify the relative contribution of the program sites to the TB prevalence. This models the logarithm of the TB prevalence (total cases over total patients enrolling in care) over time $t_{i}$. Time was expressed as temporal distance, in months, from initiation of TTS (time zero), with negative times denoting the period prior to the start of TTS i.e. 48 months prior and positive times corresponding to the period after TTS i.e. 48 months after TTS. Two different slopes, $\beta_{1}$ and $\beta_{2},$ were considered corresponding to the linear trend during the time prior to (, ${t_{i}}^{-}=\min\left(t_{i},\;0\right)$) and after TTS initiation (${t_{i}}^{+}=\max\left(t_{i},0\right)$) respectively. This effectively forces the two trend lines to meet at time zero, the time of initiation of TTS. We expect that the slopes will be significantly lower than zero, indicating that the prevalence rate ratios ($PPR_{pre-TTS}=\exp\left(\beta_{1}\right)$ and $PPR_{post-TTS}=\exp\left(\beta_{2}\right)$ across adjacent months will be significantly lower than 1. We explicitly assess whether $\beta_{1}>\beta_{2}$, i.e., whether the trend of TB prevalence after TTS is decreasing faster than prior to the inception of TTS in the model, expressed as follows:

$$\log\left(y_{i}\right)=\beta_{0}+\beta_{1}{t_{i}}^{-}+\beta_{2}{t_{i}}^{+}+\beta_{3}Age+\;\beta_{4}\;Sex+\log\left(T_{i}\right)\;\|\;Program:$$

All analyses were performed using R and STATA version 18 (Stata Corp, College Station, TX, 2015).

This analysis was approved under the EA-IeDEA Retrospective Data protocol by the Indiana University Institutional Review Board (IRB) (REC-REF 1105005572), as well as by the relevant local and national regulatory bodies affiliated with the participating programs (REC-REF IREC/2006/28 and IREC/2008/79 – AMPATH, KEMRI/RES/7/3/1 – KEMRI, NIMR/HQ/R.8a/vol.IX/440 – Tumbi, Kisesa, Morogoro, 2008-048 – IDI, MUREC 1/7 – Masaka). Regulatory bodies granted waivers for written informed consent since all data were routinely collected clinical data of public health significance that were de-identified prior to transfer to the regional data centres.

## RESULTS

**Figure 1.**
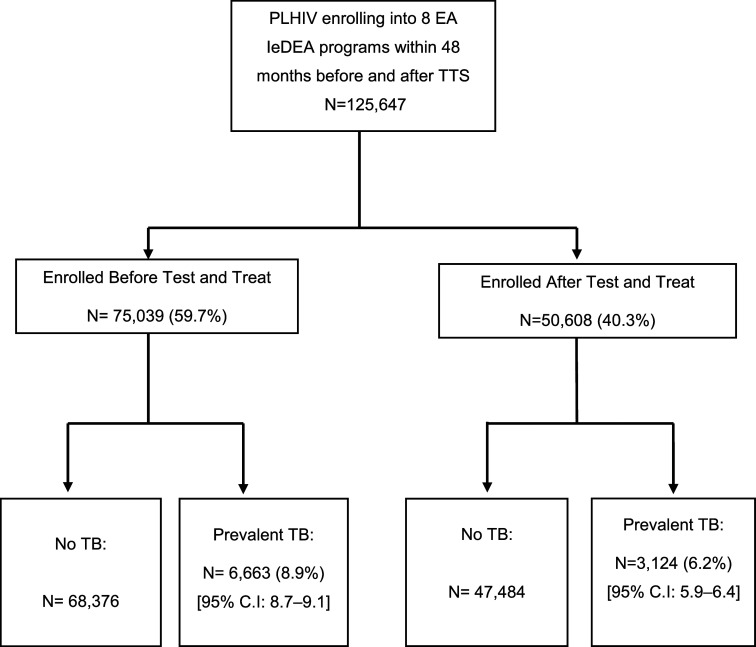
Study Flow Chart showing PLHIV enrolled into care months before and after Test and Treat strategy (TTS) implementation.

Of the 125,647 PLHIV enrolled at EA-IeDEA sites between January 2012 to December 2020, 75,039 (59.7%) were enrolled 48 months before the introduction of TTS, and 50,608 (40.3%) were enrolled after this (Figure 1). At enrolment, the median age was 32.9 (IQR: 26.8–41.0) years and the sex distribution was 37% male. The majority (52.3%) had WHO Stage 1 disease and the median body mass index (BMI) was 20.8 (IQR: 18.7–23.4) Kg/m^2^. The proportion of males enrolled prior to the adoption of the TTS was slightly lower than those enrolled after, 36.5% vs 37.9%, respectively. Median age at enrolment was similar between the two periods (32.5 (IQR: 26.6–40.5) years vs 33.5 (IQR: 27.0–41.9) years) before and after TTS adoption. BMI was also similar across the two periods being 20.7 (IQR: 18.6–23.2) versus 21.0 (IQR: 18.8–23.6) Kg/m^2^ before and after TTS, respectively. The majority of participants after adoption of TTS had WHO stage 1 disease (61% vs 48%) and initiated ART on the day of enrolment compared to before TTS, being 76% versus 23%, respectively. (Table 1).

**Table 1.** Patient characteristics at enrolment before and after Test and Treat all strategy (TTS).

Characteristic	All participants	Before TTS	Following TTS	p–value
	N = 125,647	N = 75,039	N = 50,608	
Male sex	46,564 (37.1%)	27,378 (36.5%)	19,186 (37.9%)	<0.001^A^
Age (years) median (IQR)	32.9 (26.8, 41.0)	32.5 (26.6, 40.5)	33.5 (27.0, 41.9)	<0.001^B^
Age group (years)				<0.001^A^
18–24	22,960 (18.3%)	14,065 (18.7%)	8,895 (17.6%)	
25–34	48,444 (38.6%)	29,524 (39.3%)	18,920 (37.4%)	
35–44	32,514 (25.9%)	19,212 (25.6%)	13,302 (26.3%)	
45+	21,729 (17.3%)	12,238 (16.3%)	9,491 (18.8%)	
WHO stage^E^	21,899	8,737	13,162	<0.001^A^
1	54,307 (52.3%)	31,568 (47.6%)	22,739 (60.7%)	
2	26,413 (25.5%)	18,430 (27.8%)	7,983 (21.3%)	
3	18,146 (17.5%)	13,161 (19.9%)	4,985 (13.3%)	
4	4,882 (4.7%)	3,143 (4.7%)	1,739 (4.6%)	
ART initiation^D^	17,818 (14.2%)	14,709 (19.6%)	3,109 (6.1%)	<0.001^A^
Same day	55,712 (44.3%)	17,402 (23.2%)	38,310 (75.7%)	
Within 14 days	14,205 (11.3%)	9,175 (12.2%)	5,030 (9.9%)	
within 30 days	9,806 (7.8%)	7,878 (10.5%)	1,928 (3.8%)	
within 60 days	8,401 (6.7%)	7,489 (10.0%)	912 (1.8%)	
past 60 days	19,705 (15.7%)	18,386 (24.5%)	1,319 (2.6%)	
Body Mass Index, Kg/m^2^, median (IQR)^C^	20.8 (18.7, 23.4)	20.7 (18.6, 23.2)	21.0 (18.8, 23.6)	<0.001^B^
BMI categories Kg/m^2^				<0.001^A^
<18.5	22,099 (23.0%)	13,046 (24.0%)	9,053 (21.8%)	
18.5 to <25.0	58,790 (61.3%)	33,519 (61.6%)	25,271 (60.8%)	
25.0 to <30.0	11,501 (12.0%)	6,061 (11.1%)	5,440 (13.1%)	
≥30.0	3,521 (3.7%)	1,748 (3.2%)	1,773 (4.3%)	
Prevalent TB	9,787 (7.8%)	6,663 (8.9%)	3,124 (6.2%)	<0.001^A^

A Pearson Chi-square test;

B Mann-Whitney U test.

Missing values:

C BMI [(Total= 29,736, 23.7%; Before=20,665, 27.5%; After=9,071, 17.9%);

D ART initiation (Total= 17,818, 14%; Before= 14,709 (19.6%); After=3,109 (6.1%);

E WHO stage (N= 21,899, 17%; Before=8,737 (11.6%), After=13,162 (26%).

### Impact of Test and Treat strategy on the prevalence of TB

**Figure 2.**
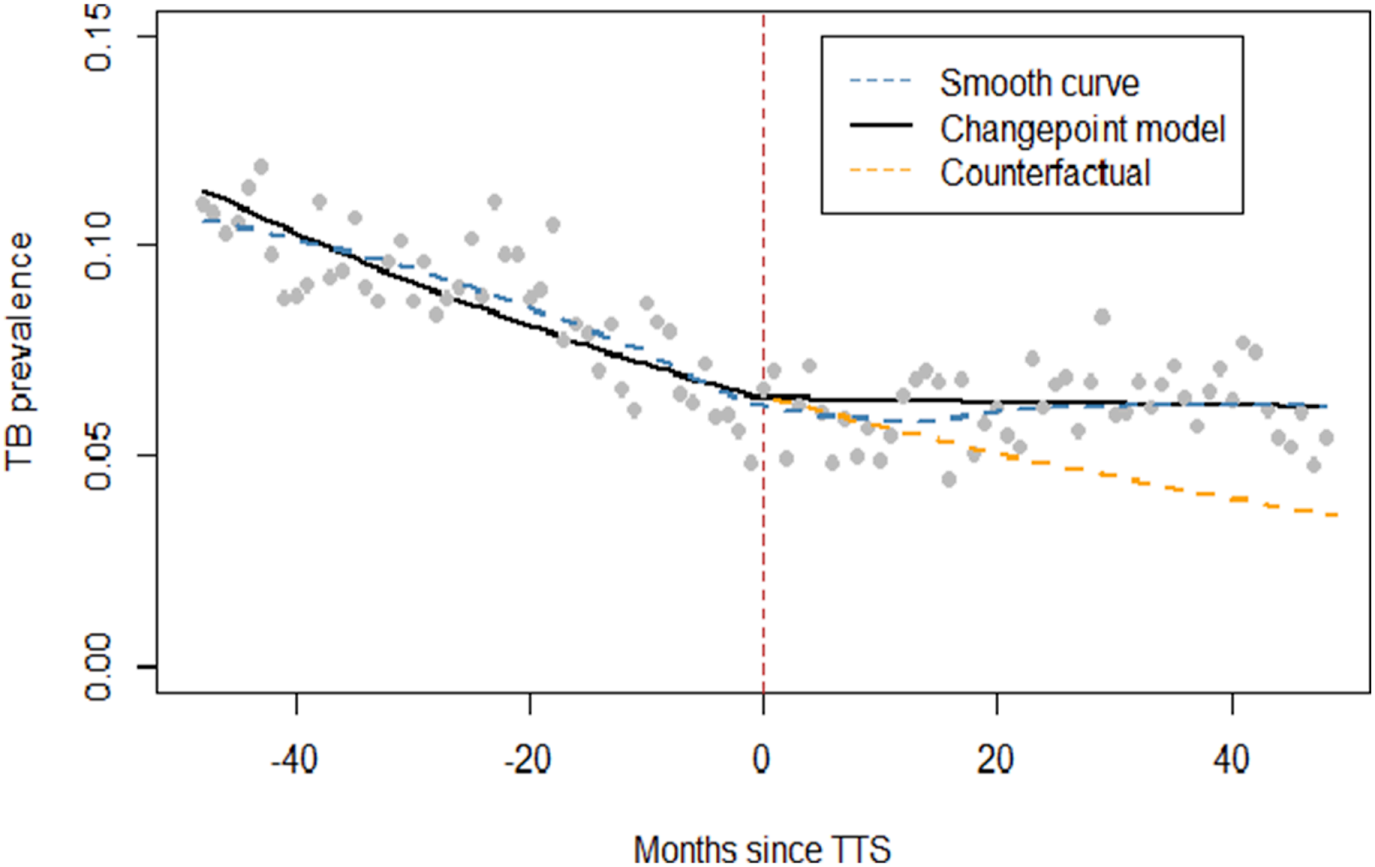
Change point model showing EA trends in TB prevalence before and after Test and Treat strategy (TTS) implementation. A smooth linear trend (blue dashed line) was added to assess model fit. The orange dashed line shows what TB prevalence would have been, had the trend of TB prevalence over time, observed prior to TTS initiation, continued after inception of the policy. The trends are shown in solid black lines in the linear scale. A smooth trend was also fit. It largely follows the linear trend posited by the model, supporting the linear structural assumption imposed by the CP model as well as the lack of any visible continued reduction in TB prevalence post TTS.

**Figure 3.**
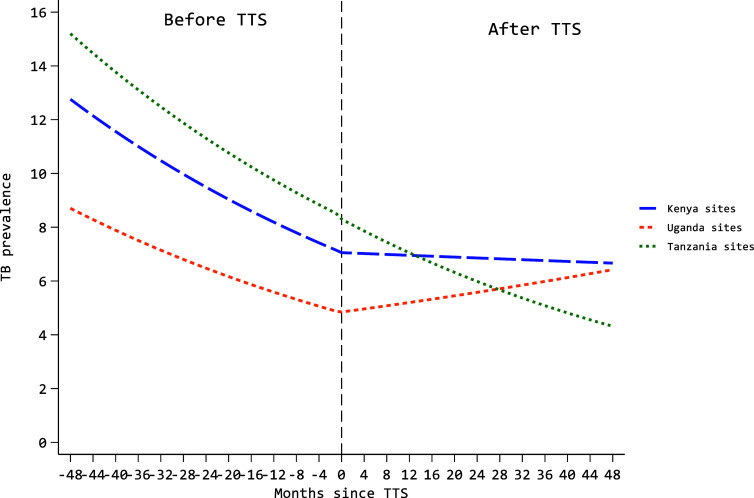
Trends in TB prevalence by country before and after Test and Treat strategy (TTS) implementation. The figure shows trends of monthly TB prevalence among PLHIV enrolled in IeDEA cohort. The dotted line indicates a point when TTS was rolled out. The prevalence of TB was on a declining trend across all three countries before TTS. Following TTS, the TB prevalence trend in Tanzania continued to decline, but plateaued in Kenya and went up in Uganda.

The prevalence of TB was lower, 6.2% (3,124) [95% C.I: 5.9–6.4] following the introduction of the TTS compared to the period before, 8.9% (6,663) [95% C.I: 8.7–9.1] (Table 1). TB prevalence differed across sex, age-groups and programs. Significant variability was noted across programs (Random intercept variance=0.275 [0.103, 0.737]; see Supplementary Data Table S1–S3). The trend analysis, adjusted for sex and age, showed a downward trend in TB prevalence, decreasing by 1.1% each month compared to the TB prevalence of the preceding month (adjusted PRR=0.989 [95%CI 0.987, 0.990], Wald test p<0.001) before TTS. On the other hand, there was no statistically significant change in TB prevalence after TTS (adjusted PRR=0.999 [95%CI 0.997, 1.000], Wald test p=0.131) that generally plateaued before a non-significant decline in the first 8-months of COVID-19 lockdowns (Table 2, Figure 2 and see Supplementary Data Table S6, Figure S1). A trend analysis, disaggregated by country, revealed variation in TB prevalence trends post-TTS, plateauing in Kenya, continuing to decline in Tanzania, but moving upward in Uganda (Figure 3). No significant time-by-sex or time-by-age interaction in TB prevalence rates was detected either before or after TTS (all p-values for interaction terms were >0.05).

**Table 2.** Rate of change in TB prevalence before and after implementation of TTS, adjusting for sex and age; estimated using mixed effects Poisson regression model.^†^

Covariate	Adjusted PRR (95% CI)	P-value
Fixed effects		
Before TTS period (per a month increase)	0.989 (0.987, 0.990)	<0.001
After TTS period (per a month increase)	0.999 (0.997, 1.000)	0.131
Random effects		
Variance across program (random intercept)	0.275 (0.103, 0.737)	

† Mixed effects Poisson regression model used to estimate rate of change in prevalence was adjusted for sex and age (18–24, 25–34, 35–44, 45–54, 55+ years). Interactions were not significant between time period and sex (interaction p-value= 0.327) or between time period and age groups (all p-values for interaction terms were >0.05).

PRR = Prevalence Rate Ratio; CI = Confidence Interval, TTS = Test and Treat Strategy

## DISCUSSION

In this descriptive study, we compare trends in the prevalence of TB before and after the adoption of TTS across 8 HIV care and treatment programs in the EA-IeDEA regional consortium. We found that among PLHIV enrolling in care, the trend in TB prevalence was in steady decline before TTS inception but plateaued after TTS. Prevalent TB was as frequently present in males and PLHIV ≥25 years in the TTS era as before. Notably, more PLHIV were in WHO stage 1, and initiated ART at enrolment after TTS compared to before suggesting high fidelity to TTS implementation. Our study findings are consistent with WHO reports showing there was a steady decline in TB prevalence globally and in Africa before TTS inception.^10^ However, our programmatic data showed that EA regional TB prevalence trend plateaued in the TTS era. Closer examination revealed that while the trend in prevalent TB plateaued regionally in the TTS era, it continued in steady decline in Tanzania, was upward in Uganda, and levelled out in Kenya – similar to published trends in the WHO Global TB report.^24^ These variations may be attributed to differences in country-specific implementation of TTS policy, TB diagnosis and treatment initiation protocols, or other concurrently run national TB control interventions. For example, in Uganda and Kenya, multiple new campaigns were launched and previously implemented interventions scaled up following TTS, leading to the observed upsurge in TB notification.^18^ These included MoH-led national campaigns for intensive TB case finding (ICF), TB-symptoms screening for all PLHIV initiating ART, improved access to molecular TB diagnostics and integrated HIV-TB care, which together contributed to higher TB case detection.^25–28^ Moreover, the introduction of superior molecular TB diagnostics in the TTS era improved TB case detection, which, along with limited changes in empiric TB treatment practices, contributed to an overall increase in notifiable prevalent TB disease.^29^ In addition, people with advanced HIV disease, among ART-naïve and -experienced PLHIV, in whom the burden of TB is high, remained significantly high after TTS.^30^ Put together, the prevalence of TB has remained high post-TTS which casts significant doubt on the adequacy of current TB control strategies to End TB by 2030.^4^

Our analyses also revealed that males, continued to have a higher burden of TB disease than females in the post-TTS era as before.^31^ While several hypotheses have been suggested for this observed phenomenon, it is still unclear whether sex differences in socio-occupational risks (smoking, mobility, socialization), health seeking behaviors, or immune profiles, play a significant role in TB disease risk^32–34^ In addition, our study also showed that TB disease was more frequently present in older PLHIV enrolling into ART care, likely due to increased exposure to TB and waning immunity with ageing.^35^ These data suggest that older PLHIV should be prioritised for earlier and more frequent TB screening, prevention, diagnosis and treatment.

Notable strengths of our study include using data collected from real-world routine HIV care settings across EA for adults, who compared to children, have a higher risk of active TB and transmission. Additionally, we avoided underestimating the burden of TB by defining TB status based on commonly used criteria applied across national ART programs that include: bacteriological, molecular, clinical and empiric TB treatment diagnoses (Supplementary Data Table S5). However, our study had some limitations. A high proportion of participants were enrolled from only one of the eight programs included in the analysis, limiting study generalizability to similar populations and care settings. Additionally, there was a potential risk of over-estimating prevalent TB since clinical or empirically treated TB is not always based on bacteriological confirmation. Moreover, data on other variables that would have been used to complete a more informative regression analysis were unavailable pre or post TTS eras (for example: changes in urbanization and population indices, country-level access to molecular testing, as well as site level data on the fidelity of TTS, uptake of TB preventive therapy, TB case finding practices). Additionally, our analysis did not account for residual confounding from other concurrent interventions implemented at the time such as integrated HIV-TB care or variations in national HIV-TB policy implementation or for the extended post-COVID-19 period.

## CONCLUSION

The decline in the prevalence of TB plateaued post implementation of TTS, likely due to improved detection overtime. Intensified TB prevention, and treatment strategies targeting males and adults ≥25 years enrolling in HIV care programs, are recommended.

## Supplementary Material



## References

[1] UNAIDS. IN DANGER: UNAIDS Global AIDS Update 2022. Geneva: Joint United Nations Programme on HIV/AIDS; 2022.

[2] World Health Organization. Global tuberculosis report. Geneva: WHO, 2015.

[3] Zhang S-X, Epidemiological features and temporal trends of the co-infection between HIV and tuberculosis, 1990–2021: findings from the Global Burden of Disease Study 2021. Infectious Diseases of Poverty. 2024;13(1):59. 39152514 10.1186/s40249-024-01230-3 PMC11328430

[4] World Health Organization. Global tuberculosis report 2024. Geneva: WHO, 2024.

[5] Ellis PK, Martin WJ, Dodd PJ. CD4 count and tuberculosis risk in HIV-positive adults not on ART: a systematic review and meta-analysis. PeerJ. 2017;5:e4165. 29259846 10.7717/peerj.4165 PMC5733368

[6] Suthar AB, Antiretroviral therapy for prevention of tuberculosis in adults with HIV: a systematic review and meta-analysis. PLoS medicine. 2012;9(7):e1001270-e. 22911011 10.1371/journal.pmed.1001270 PMC3404110

[7] Bucher HC, Isoniazid prophylaxis for tuberculosis in HIV infection: a meta-analysis of randomized controlled trials. Aids. 1999;13(4):501-7. 10197379 10.1097/00002030-199903110-00009

[8] World Health Organization. Progress report 2016: prevent HIV, test and treat all: WHO support for country impact. Geneva: WHO, 2016.

[9] World Health Organization. Global Tuberculosis Report. Geneva: WHO, 2020.

[10] World Health Organization. Global Tuberculosis Report 2022. Geneva: WHO, 2023.

[11] Gupta RK, Does antiretroviral therapy reduce HIV-associated tuberculosis incidence to background rates? A national observational cohort study from England, Wales, and Northern Ireland. Lancet HIV. 2015;2(6):e243-e51. 26423197 10.1016/S2352-3018(15)00063-6

[12] Suthar AB, Antiretroviral therapy for prevention of tuberculosis in adults with HIV: a systematic review and meta-analysis. PLoS Med. 2012;9(7):e1001270. 22911011 10.1371/journal.pmed.1001270 PMC3404110

[13] Gao J, Zheng P, Fu H. Prevalence of TB/HIV Co-Infection in Countries Except China: A Systematic Review and Meta-Analysis. PLoS ONE. 2013;8(5):e64915. 23741419 10.1371/journal.pone.0064915 PMC3669088

[14] Gelaw YA, HIV Prevalence Among Tuberculosis Patients in Sub-Saharan Africa: A Systematic Review and Meta-analysis. AIDS and Behavior. 2019;23(6):1561-75. 30607755 10.1007/s10461-018-02386-4

[15] World Health Organization. WHO conducts mid-term review of Uganda’s response to TB. Geneva: WHO, 2023.

[16] UNAIDS. Tanzania Fact Sheet, UNAIDS, Geneva: WHO, 2021.

[17] Kalema N, Gaps in TB preventive therapy for persons initiating antiretroviral therapy in Uganda: an explanatory sequential cascade analysis. Int J Tuberc Lung Dis. 2021;25(5):388-94. 33977907 10.5588/ijtld.20.0956

[18] Musaazi J, Increased uptake of tuberculosis preventive therapy (TPT) among people living with HIV following the 100-days accelerated campaign: A retrospective review of routinely collected data at six urban public health facilities in Uganda. PLoS ONE. 2023;18(2):e0268935. 36821550 10.1371/journal.pone.0268935 PMC9949662

[19] Lukoye D, Tuberculosis Preventive Therapy among Persons Living with HIV, Uganda, 2016–2022. Emerging Infectious Disease journal. 2023;29(3):609. 10.3201/eid2903.221353 PMC9973710 36823496

[20] World Health Organization. Consolidated guidelines on the use of antiretroviral drugs for treating and preventing HIV infection: recommendations for a public health approach. Geneva: WHO, 2016. 27466667

[21] Uganda MoH. Consolidated guidelines for Prevention and Treatment of HIV in Uganda. 2016

[22] Programme TURoTMoHNAC. National AIDS Control Programme, United Republic of Tanzania. National Guidelines for the Management of HIV and AIDS in Tanzania. 2016. 2016.

[23] Kenya MoH. Guidelines on Use of Antiretroviral Drugs for Treating and Preventing HIV Infection in Kenya. 2016.

[24] World Health Organization. Number of people with new or relapse episodes of TB notified per year, 2018–2023. Geneva: WHO, 2024

[25] World Health Organization. WHO High-priority target product profiles for new tuberculosis diagnostics. Geneva: WHO, 2014.

[26] World Health Organization. Systematic screening for active tuberculosis: an operational guide. Geneva: WHO, 2015.

[27] Uganda MoH. Manual for management and control of Tuberculosis and Leprosy in Uganda, 3rd Edition.; 2017.

[28] Salomon A, Interventions to improve linkage along the HIV-tuberculosis care cascades in low- and middle-income countries: A systematic review and meta-analysis. PLoS ONE. 2022;17(5):e0267511. 35552547 10.1371/journal.pone.0267511 PMC9098064

[29] Ochodo EA, Variation in the observed effect of Xpert MTB/RIF testing for tuberculosis on mortality: A systematic review and analysis of trial design considerations. Wellcome Open Research. 2019;4:173. 32851196 10.12688/wellcomeopenres.15412.1 PMC7438967

[30] Kitenge MK, Prevalence and trends of advanced HIV disease among antiretroviral therapy-naïve and antiretroviral therapy-experienced patients in South Africa between 2010-2021: a systematic review and meta-analysis. BMC Infect Dis. 2023;23(1):549. 37608300 10.1186/s12879-023-08521-4 PMC10464046

[31] Horton KC, Sex Differences in Tuberculosis Burden and Notifications in Low- and Middle-Income Countries: A Systematic Review and Meta-analysis. PLoS Med. 2016;13(9):e1002119. 27598345 10.1371/journal.pmed.1002119 PMC5012571

[32] Miller PB, Association between tuberculosis in men and social network structure in Kampala, Uganda. BMC Infect Dis. 2021;21(1):1023. 34592946 10.1186/s12879-021-06475-z PMC8482622

[33] Chaisson LH, Sex differences in tuberculosis infection and disease among people with HIV. Aids. 2025;39(2):184-192. 39453876 10.1097/QAD.0000000000004045 PMC11717608

[34] Chikovore J, Missing men with tuberculosis: the need to address structural influences and implement targeted and multidimensional interventions. BMJ Global Health. 2020;5(5):e002255. 10.1136/bmjgh-2019-002255 PMC7223013 32371568

[35] Davies LRL, Age and sex influence antibody profiles associated with tuberculosis progression. Nature Microbiol. 2024;9(6):1513-25. 38658786 10.1038/s41564-024-01678-x PMC11153143

