# Supplementary material for: Trends in prevalent TB among persons enrolling for HIV care before and after ‘Test and Treat’ across East-Africa
Source: IJTLD Open. 2025 Jun 13;2(6):359–65. doi: 10.5588/ijtldopen.24.0687 (PMC12168729; doi:10.5588/ijtldopen.24.0687)

Supplementary Table S1: Prevalence of TB 48 months before and following TTS by sex, age and program

	Before TTS	Following TTS	P-value [a]
	Prevalent TB<br>n/N (%)	Prevalent TB<br>n/N (%)	
Overall	6,636/74,272 (8.9%)	3,182/50,889 (6.2%)	*N/A*
Sex			0.117
Female	2,911/47,186 (6.2%)	1,296 (4.1%)	
Male	3,725/27,086 (13.7%)	1,886/19,304 (9.8%)	
Age group (years)			0.029
18-24	586/13,948 (4.2%)	232/8,937 (2.6%)	
25-34	2,532/29,211 (8.7%)	1,047/19,029 (5.5%)	
35-44	2,214/19,016 (11.6%)	1,106/13,379 (8.3%)	
45-54	928/8,442 (11.0%)	549/6,361 (8.6%)	
55+	376/3,655 (10.3%)	248/3,183 (7.8%)	
Program			<0.001
Uganda			
IeDEA site 1	679/3,633 (18.7%)	247/1,738 (14.2%)	
IeDEA site 2	705/7,274 (9.7%)	406/3,470 (11.7%)	
IeDEA site 3	371/14,797 (2.5%)	298/11,971 (2.5%)	
Kenya			
IeDEA site 4	3,647/32,846 (11.1%)	1,805 /25,401 (7.1%)	
IeDEA site 5	701/11,268 (6.2%)	118/2,945 (4.0%)	
Tanzania			
IeDEA site 6	127/1,309 (9.7%)	40/1,925 (2.1%)	
IeDEA site 7	191/1,698 (11.3%)	139/2,019 (6.9%)	
IeDEA site 8	215/1,447 (14.9%)	129/1,420 (9.1%)	

N = Total participants in a group, n = number of prevalent TB cases in a group, % = prevalence of TB, N/A = not applicable

[a]p-value obtained from Mantel-Haenszel test for heterogeneity of TB prevalence across the groups

Supplementary Table S2: Participant enrolment by IeDEA Program 48 months before and after TTS

East Africa (EA) Programs	All participants<br><br>N = 125647[1]	Pre-TTS<br><br>N = 75039 (59.7%)[1]	Post-TTS<br><br>N = 50608 (40.3%)[1]
Kenya			
AMPATH	58,505 (46.6%)	33,242 (44.3%)	25,263 (49.9%)
FACES	14,270 (11.4%)	11,387 (15.2%)	2,883 (5.7%)
Uganda			
IDI	5,371 (4.3%)	3,633 (4.8%)	1,738 (3.4%)
MASAKA	10,786 (8.6%)	7,332 (9.8%)	3,454 (6.8%)
RAKAI	26,857 (21.4%)	14,934 (19.9%)	11,923 (23.6%)
Tanzania			
KISESA	3,248 (2.6%)	1,325 (1.8%)	1,923 (3.8%)
TUMBI	2,880 (2.3%)	1,467 (2.0%)	1,413 (2.8%)
MOROGORO	3,730 (3.0%)	1,719 (2.3%)	2,011 (4.0%)

Supplementary Table S3: Variability (variances) across sites

Site	Variance (95% CI)
Uganda	
IeDEA site 1	0.53(0.44 – 0.62)
IeDEA site 2	0.04(0.02 – 0.07)
IeDEA site 3	1.29(1.13 – 1.46)
Kenya	
IeDEA site 4	0.03(0.02 – 0.04)
IeDEA site 5	0.09(0.05 – 0.13)
Tanzania	
IeDEA site 6	0.12(0.04 – 0.23)
IeDEA site 7	0.04(0.01 – 0.09)
IeDEA site 8	0.20(0.13 – 0.30)

Table S4: Study setting of the eight HIV care and treatment Programs

Program<br><br>(TTS start, MDY)	Country – Region	Urban/Rural settings	Main Occupation	Population Catchment area (Region)	Prevalence of HIV (Region)	PLHIV served	National TB prevalence
IDI<br><br>(01/01/2016)	Uganda - Central	Urban	Trade and Commerce, Industry, Construction,	1,875,834	8.5%	9,000	253 cases per 100,000 population
MASAKA<br><br>(12/01/2016)	Uganda – Central 1	Semi-urban	Agriculture, fishing, livestock farming	285,509	8.1%	15,500	
RAKAI<br><br>(08/01/2016)	Uganda – Central 2	Rural	Agriculture, livestock farming	518,008	5.8%	8,558	
AMPATH<br><br>(08/01/2016)	Kenya - Western	Urban and Rural	Agriculture, Trade and Commerce	8,000,000	5.0%	150,000	348 per 100,000 population
FACES (KEMRI)<br><br>(07/01/2016)	Kenya – Nyanza	Semi-urban and Rural	Agriculture, Fishing, Food processing and industrial products	1,155,574	19.3%	75,000	
KISESA<br><br>(07/01/2016)	Tanzania - Mwanza	Semi-rural (primarily rural, and peri-urban)	Agriculture, fishing, small scale businesses	54,024	7.2%	6,968	208 cases per 100,000 population
MOROGORO<br><br>(10/01/2016)	Tanzania - Morogoro	Urban and rural	Agriculture and live-stock farming	3,000,000	5.6%	13,645	
TUMBI<br><br>(10/01/2016)	Tanzania - Pwani	Semi-urban to rural	Agriculture and live-stock farming	2,500,000	5.3%	12,042	

Table S5 TB case definitions

Criteria		With TB, overall	With TB, Pre-TTS	With TB, post-TTS
Definition 1		88	71	17
Definition 2		6555	4263	2292
Definition 3		9,161	6246	2915
Definition 4		962	350	612
Definition 5		711	479	232
Definition 6		95	74	21
Definition 7		677	430	247
**Total TB cases**		**9787**	**6663**	**3124**
**Diagnostic test method used**				
**Diagnostic**	**Tests done**	**Bacteriologically confirmed positive**		
TB LAM	182	10	0	10
Culture	1615	629	498	131
AFB	15	5	5	0
GeneXpert	679	224	55	169
		**868**	**558**	**310**

Note:

Definition 1: The patient started treatment within 240 days (8 months) prior to HIV care enrolment and has no evidence of successfully completing treatment prior to enrolment

Definition 2: The patient started or completed treatment within 60 days after enrolment

Definition 3: The patient is on treatment, was diagnosed with pulmonary or extrapulmonary TB, or had a positive test (ZNstain or MTB finding or AFB culture) during the 60 days after enrolment.

Definition 4: The patient is on treatment continuation phase 61-90 days after enrolment

Definition 5: The patient stopped TB treatment 61-240 days after enrolment with evidence of treatment completion (i.e. cured & smear negative, completed & no /unknown smear status)

Definition 6: The patient was diagnosed with TB within 240 days prior to enrolment and has no evidence of successfully completing treatment prior to enrolment.

Definition 7: The patient is diagnosed if had a positive test (ZNstain or MTB finding or AFB culture) during the 60 days after enrolment.

In the dataset, there were a total of 307 prevalent TB cases diagnosed with extrapulmonary TB (204/75039, 0.3%) pre-TTS and (103/50608, 0.2%).

Data on resistance were not available.

Prevalent TB trend during Covid-19 pandemic lockdown period

Table S6: Rate of change in TB prevalence before, after implementation of TTS and Covid-19 period (1st March 2020 - 31st Dec 2020) using a Poisson regression model

Covariate	PRR (95% CI)	P-value
Fixed effects		
Before TTS period (per a month increase)	0.989 (0.987, 0.990)	<0.001
After TTS period (per a month increase)	0.999 (0.997, 1.000)	0.630
Covid-19 period (per a month increase)	0.983 (0.967, 1.000)	0.061

PRR – Prevalence Rate Ratio, CI – Confidence Interval, TTS – Test and Treat Strategy

The TTS slope after Covid-19 compared to slope before (TTS), the TB prevalence declined by 1.69% (difference in slopes -.016, 95% CI: -0.035 to 0.001) of the preceding month, however, not statistically significant (p=0.070).

Figure S1: Graph showing trends of monthly TB prevalence among PLHIV before TTS, during TTS and COVID-19 period.

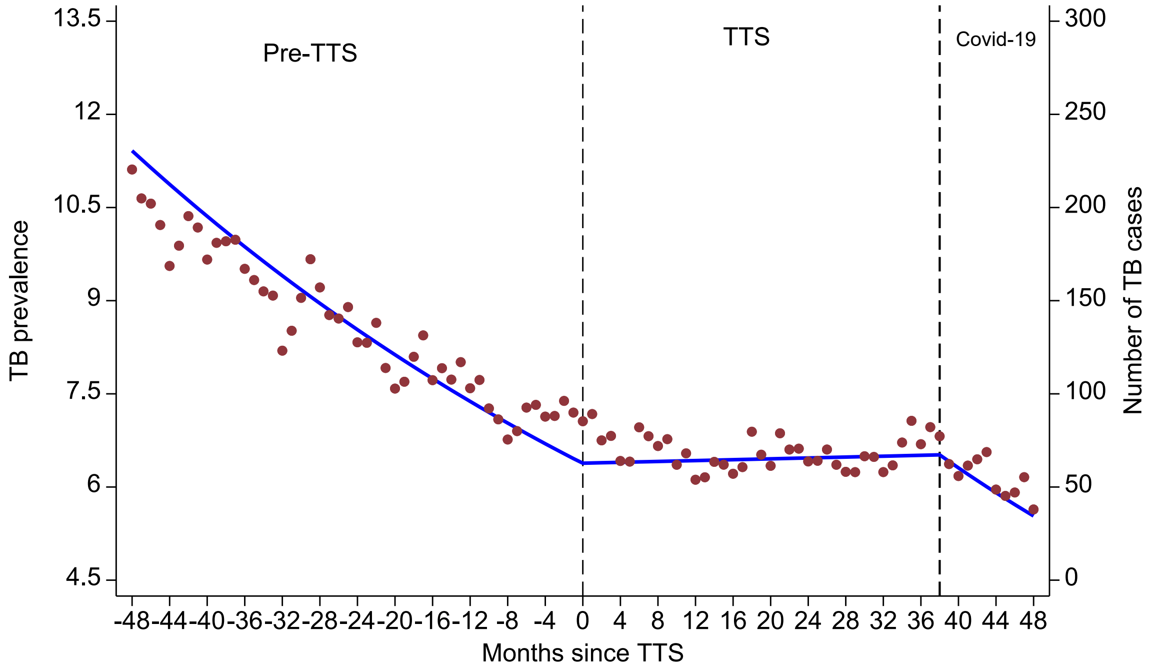

Supplement: Supplementary file 1 [file ijtldopen24-0687_supplementarydata1.pdf]
